# Nuchal Translucency and Congenital Heart Defects

**DOI:** 10.2174/011573403X264963231128045500

**Published:** 2024-01-24

**Authors:** A. Sofia-Gonçalves, L. Guedes-Martins

**Affiliations:** 1 Instituto de Ciências Biomédicas Abel Salazar, University of Porto, 4050-313, Porto, Portugal;; 2 Centro de Medicina Fetal, Medicina Fetal Porto - Centro Materno Infantil do Norte, 4099-001, Porto, Portugal;; 3 Departamento da Mulher e da Medicina Reprodutiva, Centro Hospitalar Universitário do Porto EPE, Centro Materno Infantil do Norte, Largo Prof. Abel Salazar, 4099-001, Porto, Portugal;; 4 Unidade de Investigação e Formação – Centro Materno Infantil do Norte, 4099-001, Porto, Portugal;; 5 Instituto de Investigação e Inovação em Saúde, Universidade do Porto, 4200-319, Porto, Portugal

**Keywords:** Ultrasound, nuchal translucency, multifactorial etiology, congenital heart defect, adverse pregnancy outcome, normal karyotype

## Abstract

Nuchal translucency comprises a temporary accumulation of fluid in the subcutaneous tissue on the back of a fetus’s neck, which accompanies the crown-rump length and is observed through an ultrasound performed between 11 and 13 weeks + 6 days gestation. Nuchal translucency is considered to be above normal when values are higher than the 95^th^/99^th^ percentile or equal to or higher than 2.5/3.5 mm. The first connection between increased nuchal translucency and the presence of congenital heart defects is described in the study of *Hyett et al.*, who observed that they are directly proportional. Since that time, several studies have been conducted to understand if nuchal translucency measurements can be used for congenital heart defect screening in euploid fetuses. However, there is great variability in the estimated nuchal translucency cutoff values for congenital heart defect detection. The purpose of this review was to understand how increased nuchal translucency values and congenital heart defects are related and to identify which of these defects are more frequently associated with an increase in these values.

## 1 INTRODUCTION

Nuchal translucency (NT) comprises a temporary accumulation of fluid in the subcutaneous tissue on the back of a fetus’s neck [1], which accompanies the crown-rump length (CRL) [2]. It is observed through an ultrasound performed between 11 and 13 weeks + 6 days gestation [1]. NT is considered to be above normal when values are higher than the 95^th^/99^th^ percentile or equal to or higher than 2.5/3.5 mm (if in metric units) [3]. Evaluating NT through an ultrasound evaluation is already part of the usual first-trimester screening exams for chromosomal abnormalities (CAs), namely, Down syndrome [4].

Between the 10^th^ and 14^th^ weeks of gestation, several physiological changes occur in a fetus [5]. Usually, NT increases in the 10^th^ week, reaches its highest value at approximately the 13^th^ week and starts decreasing from the 14^th^ week onward [6]. After the 10^th^ week, there is diastolic flow from the umbilical artery, which often remains incomplete until the 13^th^ week [7]. In this way, it seems that there is increased vascular resistance in the uteroplacental circulation [5]. In contrast, the decrease in the umbilical pulsatility index, which occurs during the second trimester, is synonymous with a quick decrease in vascular resistance [7]. In addition, it is noticeable that there is a change in the speed of the flow of blood in the ductus venosus (DV) between the 10^th^ and 13^th^ weeks, which abruptly increases shortly after [8]. In this way, the decrease in NT that is usually seen from the 14^th^ week onward may be due to placental impedance [5, 9] and to the start of the development of the kidney function [9].

In fetuses with normal karyotype, the increase in NT also increases the risk of adverse perinatal outcomes, namely, intrauterine fetal demise, structural birth defects, skeletal dysplasia (SkD) and genetic syndromes (GSs) [10]. Despite this, most of these fetuses have a positive outcome [10]. The incidence of CAs when NT is increased varies between 19.8% and 40% [2]. CAs correlate with fetal NT thickness in the sense that the odds increase as NT thickness increases [2]. The most common CAs associated with increased NT are trisomy 21 (T21, Down syndrome), trisomy 13 (T13, Palau syndrome), trisomy 18 (T18, Edward syndrome), monosomy X (Turner’s syndrome, TS) and triploidy [11]. T21 is the most common aneuploidy, with TS being the second most common [11].

How the pathophysiological mechanism of NT relates to the variety of associated fetal malformations with different levels of severity still needs to be determined [10].

It is known that the etiology of increased NT is strongly connected to development anomalies of the lymphatic and cardiovascular systems; however, it is highly likely that this etiology is more complex and multifactorial [4].

The genetic abnormalities underlying the mechanisms that result in the formation of nuchal edema are still mostly unknown [10]. A 2015 study identified 15 candidate genes that are probably involved in abnormal cardiovascular and lymphatic development through endothelial dysfunction [10]. A more recent study from 2022 aimed to understand how the genetic alterations observed in mutant mice could be useful in establishing the diagnosis and prognosis of human fetuses with increased nuchal translucency [12]. However, it remains to be seen whether the same genetic correlation is found in humans and how the implementation of genetic screening tests could be useful in predicting disease in fetuses with increased nuchal translucency [12].

Congenital heart defects (CHDs) constitute 30% of congenital fetal malformations, with a prevalence of 8 in 1000 live births [13]. They are considered severe when they result in fetal death or when they require surgery within the first year of life [13] to avoid permanent morbidity or death [14]. It is estimated that these occur in a proportion of 4-7:1000 live births [13]. Among all deaths due to congenital fetal malformations, CHDs account for 40% of perinatal deaths and 60% of postnatal deaths [15].

The first connection between increased NT and the presence of CHDs is described in the study of *Hyett et al.*, who observed that they are directly proportional [16]. Since that time, several studies have been conducted to understand if NT measurements can be used for CHD screening in euploid fetuses [17]. Cardiac malformations detected in the postnatal period are among the worst malformations, both in severity and prognosis [17]. Currently, we know that prenatal diagnosis only occurs in between 15% and 30% of cases [18]. The goal of detecting CHDs early is to allow for early care, with the aim of better outcomes [19].

There is great variability among studies regarding estimated NT value cutoffs for CHD detection [15]. According to a meta-analysis, when the cutoff is in the 95^th^ percentile, sensitivity is 37%, which decreases to 31% when a cutoff in the 99^th^ percentile is used [15]. A recent study determined a specificity of 93.8% for the 95^th^ percentile [20], whereas the 99^th^ percentile showed a specificity of 99.0% [20, 21]. Despite this, guidelines from the *American College of Obstetricians* and *Gynecologists* recommend performing a fetal echocardiogram every time the NT value is equal to or superior to 3.5 mm [14]. On the one hand, ultrasound has limited efficiency (from 17% to 60%) in detecting CHDs in random fetuses [22]. On the other hand, current scientific evidence states that approximately 35% to 40% of CHDs are diagnosed in fetuses classified as being at low risk [13]. It is not yet clear what is the best cutoff for NT screening [19].

The purpose of this review was to understand how increased NT values and CHDs are related and to identify which of these defects are more frequently associated with an increase in these values.

## 2 MATERIALS AND METHODS

### 2.1 Information Sources and Search

The search was performed using the PubMed database to identify relevant articles. The first search occurred on August 14^th^, 2022, and the last search occurred on March 20^th^, 2023. To optimize the search, the MeSH Database was used. The MeSH terminology used was ‘Nuchal Translucency Measurement,’ ‘Heart Defects, Congenital/physiopathology’, ‘Heart Defects, Congenital/diagnosis’, ‘Heart Defects, Congenital/epidemiology’, ‘Prenatal Diagnosis,’ ‘Pregnancy Trimester, First’ and ‘Pregnancy outcome.’

**Fig. (1):**
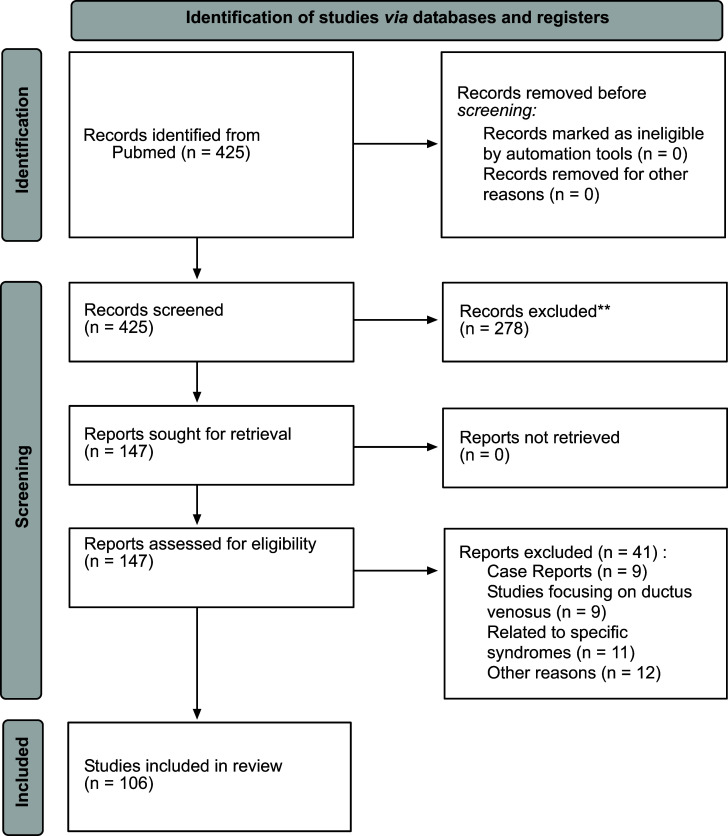
PRISMA 2020 flow diagram of study selection.

Another strategy adopted was using PubMed's Advanced Search Builder with the keywords ‘increased nuchal translucency,’ ‘congenital heart defects’ and ‘normal karyotype.’ The search included the bibliographic references listed in the most interesting articles, and similar article functionality from the PubMed database was also used. This revision was made in accordance with the PRISMA 2020 guidelines (Fig. **1**).

### 2.2 Eligibility Criteria

Study inclusion was based on the following criteria: Increased NT, based on ultrasounds, the diagnosis of at least one CHD, and the exclusion of chromosome abnormalities in the fetuses. The following kinds of articles were excluded: case reports and conference abstracts. No language restrictions were applied, and only full articles were included.

### 2.3 Study Selection

Studies were selected through a two-step process. In the first step, articles were selected based on their title and abstract. In the second step, the full text was read to determine if the article should be included or excluded.

### 2.4 Data Collection Process

The following information was collected in all included studies: NT values measured during ultrasounds (the absolute value or percentile for the fetus’s age) and the type of CHD detected.

### 2.5 Outcome Measure

The outcome measure was the percentage of fetuses with CHDs that were diagnosed in the prenatal period after the detection of increased NT on the first-trimester ultrasound. Different cutoffs were considered to define NT as increased, according to the methods adopted by each study.

## 3 NORMAL AND ABNORMAL NUCHAL TRANSLUCENCY: UNDERLYING ETIOLOGY

### 3.1 Normal Nuchal Translucency

**Fig. (2):**
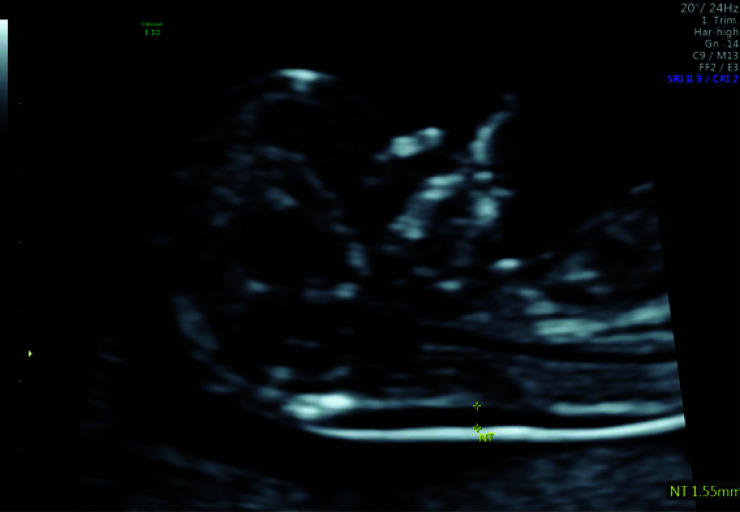
A mid-sagittal section of a fetus in the first trimester showing normal nuchal translucency.

The fluid-filled space between the back of the fetal neck and the overlying skin is called nuchal translucency [1]. It is measured in fetuses with a crown-rump length (CRL) of 45 - 84 mm [2], which correlates with gestational ages of 11 + 0 to 13 + 6 weeks [1]. NT is considered to be increased if the values are greater than the 95^th^ / 99^th^ percentile or equal to or greater than 2.5 / 3.5 mm (Fig. **2**) [3].

NT physiology is not yet completely understood [23]. For the most part, this is a result of the large gap in the knowledge of every process related to fetal development in the first 14 weeks of gestation [24].

Existing studies suggest that NT can result in transitory cerebral overperfusion (CO) and/or lymph overproduction [24, 25]. CO can result in incomplete development of the bone component that forms the posterior fossa, which plays a role in protecting the cervical region of the neural tubule [24]. Between the 9t^h^ and 12^th^ weeks of gestation, there is an increase in the volume of circulating blood, which follows the quick growth of the placenta [26, 27]. During this period, organogenesis is ongoing, and thus, protecting neuronal structures from CO is paramount, which can result in transitory, physiological nuchal edema [24].

Beyond this, the association between CHDs and increased NT alerts to the possibility of hemodynamic adjustments that occur during normal cardiovascular development and are responsible for NT [24, 28].

There are very few studies on the basic physiology of NT, and most of them have been conducted for more than 15 years, which showcases how difficult it is to understand the underlying mechanisms.

### 3.2 Abnormal Nuchal Translucency

**Fig. (3):**
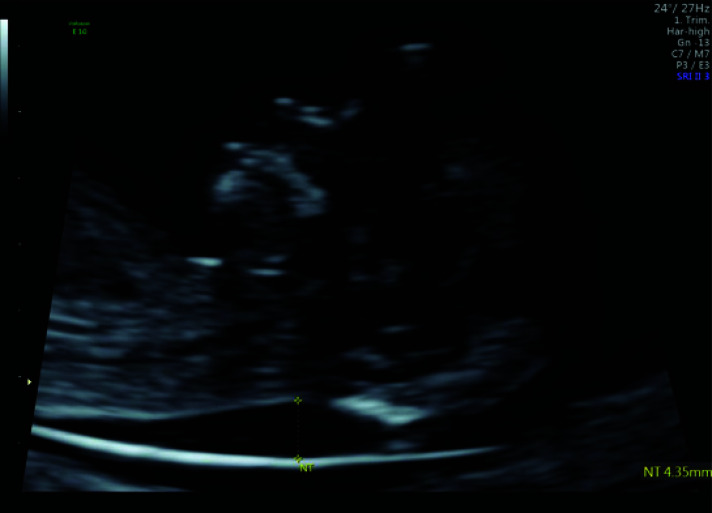
A mid-sagittal section of a fetus in the first trimester showing increased nuchal translucency.

Increased NT can be present in a variety of conditions, which suggests that its origins can be multifactorial [29]. Of the mechanisms that are most likely related, the highlights are cardiovascular anomalies and cardiac dysfunction, development disorders or lymph drainage and altered composition of the extracellular matrix [29]. These and other potentially involved mechanisms are discussed below (Fig. **3**).

#### 3.2.1 Cardiovascular Anomalies and Cardiac Dysfunction

Heart failure (HF) seems to be a deciding factor in the etiology of increased NT, as there is a relationship between increasing NT values and the presence of CHDs, both for aneuploid fetuses and euploid fetuses [30]. On the one hand, some fetuses diagnosed with any trisomy but without CHDs do not show increased NT [4, 24]. On the other hand, chromosomally normal fetuses with CHD show increased NT [4, 24]. However, some studies have shown that there is no meaningful difference between fetuses with and without increased NT, pertaining to the types of detected cardiac defects, percentage of the left ventricular ejection fraction and cardiothoracic index [29].

The placenta plays an important role as a source of oxygen and nutrients for the fetus and as a selective barrier to xenobiotics [31]. Abnormal hemodynamics in the umbilical-placental circulation can affect cardiac development [31]. Since this circulation is established late in the period of cardiac organogenesis, placental problems are more likely to biomechanically influence cardiac differentiation later in pregnancy when the placenta receives approximately 40% of the fetal cardiac output [31]. This may explain why, in the first trimester, most fetal heart defects do not develop heart failure and fetal tissue edema.

In contrast, a study showed a decrease in diastolic cardiac function in the 2nd trimester in fetuses without chromosome abnormalities but with increased NT [32]. With this in mind, we can see that the impact of cardiac dysfunction on NT physiology is not yet well understood [29].

Through molecular biology studies, we have come to understand that atrial natriuretic peptide and brain natriuretic peptide mRNA levels are increased in fetuses with trisomy and increased NT [28, 33, 34].

On the one hand, the increase in these peptides can reflect the myocardium’s normal response to fluid retention [35]. On the other hand, it is known that increasing natriuretic peptide levels are associated with HF in the prenatal period [29, 36]. Comparing both situations, we can state that fetuses with trisomy and increased NT suffer from HF, which can be a consequence of aortic isthmus (AI) narrowing [36]. *Hyett et al.* found that this narrowing may be part of the etiology of increased NT in fetuses with T13/18/21 and TS [37]. The larger the narrowing of the AI is, the thicker the NT [36].

Another study suggests that an obstruction of the aortic arch (AA) leads to preferential blood flow to fetuses’ heads and necks, which become hyperfused and, in turn, causes subcutaneous edema [36, 38]. As gestation progresses, the development of the AA and hemodynamic adaptation explain the transient effects of increased NT [36, 38]. Given that the AI is located immediately after the start of the left subclavian artery, the presence of edema in the upper limbs is also expected [38]. However, this is a rare occurrence in fetuses with increased NT [38].

Edema is a very typical sign of HF in adults; given that it is only present in the nape of fetuses with trisomy, it does not support the HF hypothesis [36, 38]. Beyond this, in adults with HF, there is a lower expression of the gene that encodes the sarcoplasmic reticulum ATPase of myocytes, which is in opposition to the unchanged genetic expression in these fetuses [29, 39, 40].

Another factor contributing to the classification of HF as an etiological factor is the abnormality of the flow of blood in the DV in fetuses with increased NT and CAs or CHD [41]. Additionally, there are several reasons why a slight decrease in diastolic cardiac function in fetuses can lead to increased NT and affect the flow in the DV: Fetal ventricles are less capable of being filled with blood during diastole; the final diastolic pressure in fetuses is higher to that in adults due to increased strain tension; in the first trimester, placental vascular resistance is still high, so the afterload is considerably higher, and the absence of kidney maturity in young fetuses leads to fluid retention [29].

#### 3.2.2 Development Disorders or Lymph Drainage

In the past, it was thought that lymphatic vessels (LV) originate in veins, which disconnect later on [25, 29]. The only exception would be the axillary jugular sacs, which maintain the bridge between the lymphatic system and the venous system to allow for lymph drainage [25, 29]. Nowadays, scientific evidence states that LV develops independently from the blood vessels (BV) [42]. Only lymphovenous anastomosis can originate in venous vessels [42]. Taking this physiology into consideration, the delay in a connection being established between both systems is a possible explanation for increased NT [29, 37]. This results in a dilation of the jugular lymphatic sacs [29, 37]. This hypothesis explains the typical temporary increase in NT [39]. Despite this, a significant difference between the pulsatility indices of the jugular vessels of fetuses with and without increased NT cannot be measured [39].

The anomalous proliferation of LV can also harm the flux of lymph [29]. A study discovered that fetuses with Turner’s syndrome show lymphatic hypoplasia, contrasting with fetuses with trisomy, who show many dilated LV that are uniformly distributed throughout the dermis and hypodermis [37, 43]. It is also possible to identify lymphatic hypoplasia with decreased lymphatic drainage in euploid fetuses with increased NT, namely, in those with Noonan’s syndrome and congenital lymphedema [29].

Another possible explanation for increased NT consists of decreased lymph drainage due to hypokinesia or fetal akinesia, which is seen in several congenital neuromuscular disturbances, such as spinal muscular atrophy, fetal akinesia deformation sequence and myotonic dystrophy [29].

Several studies appear to suggest abnormal endothelial differentiation in the physiopathology of increased NT, with it being a common denominator, together with cardiovascular and hemodynamic disturbances, which are frequently found [37]. However, endothelial defects, at least partially, can also be a result of the presence of excess nuchal fluid [35].

Studies focused on the genetic expression of lymph vessel cells in fetuses with increased NT suggest that a loss of identity can occur in lymph vessels, closing in on the phenotype of blood vessels [44]. From the many changes that occur in lymph cells that lead to increased NT, the highlights are as follows: decreased expression of the lymph markers Prox-1 and podoplanin [45]; expression of vascular endothelial growth factor A (VEGF-A) and neuropilin-1, which are typical properties of BV [45]; the presence of anomalous smooth muscle cells in the dilated jugular lymph sac [45]; increased expression of Sonic hedgehog (Shh), which, in combination with VEGF-A, promotes abnormal lymph differentiation [44]; increased expression of platelet-derived growth factor (PDGF)-B, which favors the recruitment and proliferation of smooth muscle cells; and decreased expression of the forkhead transcription factor FOXC2, whose role is to inhibit the expression of PDGF-B [44].

#### 3.2.3 Altered Composition of the Extracellular Matrix

The constitution of the extracellular matrix (EM) incorporates mucoproteins, mucopolysaccharides and collagenous fibers [29]. Integrins, such as laminine and fibronectin, are responsible for connecting cells and pinning them to the matrix [29].

Chromosomes 13, 18 and 21, often associated with trisomies, contain genes that encode many of the matrix’s proteins [29]. A few examples include COL4A1 and COL4A2 on chromosome 13, LAMA1 and LAMA3 on chromosome 18 and COL6A1 and COL6A2 on chromosome 21 [29]. In this way, changes in the EM observed in fetuses with trisomy result from the gene dosage effect [29]. Fetuses with T13 show an EM that is rich in collagen type IV; fetuses with T18 have an abundance of laminine; and fetuses with T21 show an overwhelming amount of collagen type VI [29, 46]. In addition, it is noticeable in these last examples that an increase in hyaluronic acid is responsible for pinning great quantities of solvent in the matrix [47].

In the same line of thought, fetuses with GSs can also display increased NT, which goes in hand with changes in collagen metabolism or fibroblast/peroxisome growth factor signaling [29]. Examples include achondrogenesis type II, achondroplasia and Zellweger syndrome [29].

A study showed that fetuses with increased NT present subexpression of several genes, such as fibronectin 1, endothelin 3 and tissue inhibitor of metalloproteinase 1, which are all part of the biosynthesis of the EM [39].

It is also known that the EM plays an important role in cellular migration, so changes in its constitution can be reflected in the anomalous development of several organs [48].

Despite this, changes in the EM seem insufficient as a single etiological mechanism of increased NT and do not explain temporary NT [36].

#### 3.2.4 Venous Congestion in the Head and Neck

Increased NT can also be seen in situations involving venous congestion in the head and neck due to the increased difficulty of venous return. Some examples include congenital diaphragmatic hernia, esophageal atresia [36], amnion ruptures [29] or a narrow chest due to SkD [49].

In congenital diaphragmatic hernia, abdominal viscera passes into the interior of the thorax, causing an increase in pressure [50]. Dilation of the blind extremity of the esophagus, which is seen in esophageal atresia, is responsible for the compression of BV in the head and neck [49]. Last, in SkD, intrathoracic pressure is increased, resulting in anomalous development of the thorax [29].

#### 3.2.5 Anemia

Fetuses with anemia present hyperdynamic circulation, which may be the origin of increased NT and, ultimately, may provoke fetal hydrops [51]. Fetal hydrops, which occur when there is a hemoglobin deficit above 7 g/dL [51], can have an immune or nonimmune etiology [29]. In the isoimmunization of red cells, an increase in NT values cannot be observed, given that the immaturity of the reticuloendothelial system conditions the lack of the destruction of these erythrocytes in the initial phase [29]. When anemia has a genetic cause (thalassemia, Fanconi anemia, Blackfan-Diamond anemia, *etc.*) or is secondary to a congenital infection, an increase in NT values is frequently observed [29].

#### 3.2.6 Infection

The presence of maternal infection does not imply the presence of fetal infection, even if the fetus has increased NT [29]. Beyond this, the incidence of infection in fetuses with increased NT in the first trimester and normal karyotype is similar to that of the general population [29]. Thus, screening for maternal infection is not advised when chromosomally normal fetuses present increased NT [29, 36]. Screening should be performed only when a fetus develops nape edema in the second/third trimester or when generalized hydrops are observed [29, 36].

Parvovirus B19 infection is the only infection with a proven association with increased NT [29]. Its physiopathological basis consists of the progression to HF and suppression of hematopoiesis, which conditions the development of fetal anemia [36].

#### 3.2.7 Hypoproteinemia

It has been noted that fetuses with congenital nephrotic syndromes, such as the Finnish type of congenital nephrotic syndrome and diffuse mesangial sclerosis, can show increased NT in the first trimester. The most likely physiopathological mechanism underlying this is hypoproteinemia, which is an outcome of proteinuria [49-55].

## 4 PREGNANCY OUTCOMES OF ABNORMAL NUCHAL TRANSLUCENCY

Increased NT can be interpreted as an indicator that something might be compromising the health of a fetus and that there is a risk of an APO [56, 57]. The association between increased NT and T21 is well known, but it can be related to other Cas [58]. Beyond this, increased NT may be present in fetuses with an NK and can be associated with a considerable variety of structural abnormalities (SA) and GSs [58]. However, it is difficult to establish a clear association between increased NT and GSs, given that these are often rare, and their simultaneous presence can simply be a coincidence [58, 59].

The outcome that most frequently relates to increased NT is the presence of CHDs [60, 61]. Fetuses with increased NT have a relative risk of developing a CHD that is 6.6 times higher than that of fetuses with normal NT [62]. The same study revealed a prevalence of CHDs in 6% of fetuses with an NT value above 3 mm and 20% of fetuses with an NT value above 5.5 mm [63]. A study by the Fetal Medicine Foundation (FMF) concluded that fetuses with NT values above the 99^th^ percentile have an increased risk of developing a major CHD that is at least 10x higher than that of the general population [55].

The incidence of CAs is as high as the increase in NT [60, 64]. When an NT value is higher than the 3 mm cutoff, the rate of CAs varies between 16.6% and 48% [64] and increases to 75% for NT values above 5 mm [65]. Approximately 78% of fetuses with T18 [64] and 70% of fetuses with T13 [57] have NT values above the 95^th^ percentile. Increased NT is also frequently present in other kinds of CAs, with a few examples being TS, triploidy [57], and other less frequent aneuploidies, such as trisomy 10 [66, 67].

The higher the NT value is, the greater the risk of in-utero death (IUD), spontaneous abortion (SAb) and SA (that can interrupt pregnancy and cause postnatal death or life with deficiencies) [58]. The likelihood of an APO for a fetus with increased NT is 32% if the NT value is between 3.5 and 4.4 mm, 49% if the NT value is between 4.5 and 5.4 mm, 67% if the NT value is between 5.5 and 6.5 mm and 89% if the NT value is equal to or above 6.5 mm [58]. In fetuses that are chromosomally normal with increased NT, there is also an increased incidence of SAb and IUD [57, 68, 69] . In this way, when increased NT is detected in a chromosomally normal fetus, the parents should receive adequate information to make an informed decision regarding the pregnancy [70, 71].

Older studies showed no relationship between increased NT and preeclampsia, fetal growth restriction, premature birth and low birth weight [57]. However, a 2018 study demonstrated a slight but significant increase in these outcomes in these fetuses [72]. Additionally, this same study did not present significant differences related to the Apgar score, premature placenta separation and gestational diabetes [72].

In general, in 70 to 90% of pregnancies with fetuses with increased NT, there is a chance that the fetus will develop without any issues [56].

It is difficult to estimate the prevalence of neurodevelopmental problems in fetuses with increased NT, given that their manifestation can occur in a variable period of time after birth [73]. In this way, it is likely that this prevalence is underestimated [73]. In two older studies, the prevalence of neurodevelopment delays in children who showed increased NT during their fetal period was between 3.2 and 5.6% [68, 74]. In a more recent study, the prevalence obtained was 1.2% for children with increased NT and 0.4% for children with NT within the expected values [58]. However, all of these studies were conducted over 20 years ago.

An increasing number of GSs have been associated with increased NT in the first trimester [57]. Currently, this number is beyond 150, with some examples being Noonan syndrome, type II achondrogenesis and Smith-Lemli-Opitz syndrome [75].

A 2001 study documented a higher prevalence of allergic symptoms to food, animals, antibiotics and pollen in children who had increased NT during pregnancy [76].

In gestations of monochorionic twins (MT) in which one of the fetuses has increased NT, it is necessary to consider the higher chance of another APO, namely, twin-to-twin transfusion syndrome (TTTS) [57]. A study determined that the risk of TTTS is, on average, 3 times higher in these twins when compared to MT with normal NT [77].

## 5 CUTOFF LEVELS OF NUCHAL TRANSLUCENCY AND ABNORMAL CARDIAC FINDINGS

The diagnosis of a CHD is made approximately 6 weeks earlier in fetuses who have an increased NT compared to those with NT within the standard range [78]. However, there is a large heterogeneity in the results among the existing studies regarding the best cutoff point for NT in the detection of CHDs [79, 80]. This difference in the results is currently not fully understood and may derive from chance or an unidentified residual bias [79, 80].

However, a better understanding of the most effective NT cut-off in the early detection of CHDs requires interpretation, comparison and discussion of the currently existing scientific evidence [79, 80]. Therefore, we will provide an overview of the results of studies performed between 1985 and 2013, keeping in mind that all of these studies included only fetuses with NK [17, 19-22, 62, 69, 81]. Beyond this, a strategic split between scientific evidence obtained up to 2002 [20, 22, 69, 81-85] and scientific evidence obtained up to 2013 [19, 21, 62, 69, 86-99], along with a split between studies in which NT was and was not measured by FMF-certified operators [20, 69, 86, 86-95, 99], up to 2013, made it easier to conclude. The main parameters evaluated were sensitivity and specificity, taking into consideration that some studies used the 95^th^ and 99^th^ percentiles as cutoffs, while others opted to use absolute values in millimeters [79, 80].

### 5.1 Scientific Evidence up to 2002

Considering 8 studies conducted up to 2002, CHDs were detected in 162 fetuses of the 58492 studied (0,28%) [20, 22, 69, 81-85]. Using the 95^th^ percentile cutoff (2.2-2.8 mm, or alternatively, ≤ 3 mm), the sensitivity was 37% (95% CI, 25%-51%), and the specificity was 96.6% (95% CI, 95,1%-97,6%) (Table **1**) [80].

Out of these eight studies, only six also used the 99^th^ percentile (or ≥ 3.5 mm, or alternatively, between 3-5 mm) as a cutoff for NT [21, 23, 70, 82, 84, 86]. In this case, CHDs were diagnosed in 139 fetuses of a total of 49804 fetuses [20, 22, 69, 81-83]. The sensitivity and specificity were 31% (95% CI, 18%-49%) and 98.7% (95% CI, 98,1%-99,2%), respectively [80].

The inherent positive predictive value (PPV) of an NT cutoff reflects the probability of a fetus having a CHD [80]. It was noted that for a CHD rate of 0.28%, a cutoff of the 95^th^ percentile (or equivalent absolute values, as previously mentioned) showed a PPV of 3%, while a cutoff of the 99^th^ percentile (or equivalent absolute values) showed a PPV of 6% [80].

From a more practical perspective, it is important to know how many fetuses sent for fetal echocardiography following the detection of increased NT truly had a CHD [80]. The cutoff of the 95^th^ percentile allows for the diagnosis of one fetus with a CHD among 33 suspected fetuses, while a cutoff of the 99^th^ percentile allows for the diagnosis of one in 16 suspected cases [80, 82].

### 5.2 Scientific Evidence up to 2013

Since 2002, there have been changes with respect to the resolution of the echographs and the methods of measuring NT [79]. Moreover, new studies have been published, and thus, the current knowledge about how increased NT and CHDs are related is vaster [79, 80]. With this in mind, the need for a new way to evaluate the role of increased NT in early CHD detection in fetuses has emerged (Table **2**) [79].

In total, 17 studies that used the 95^th^ percentile cutoff were evaluated, corresponding to 151369 fetuses, which presented a proportion of CHD of 2.8:1000 [19, 20, 62, 86-98]. The calculated sensitivity was 44.4% (95% CI, 39.5-49.5), while the specificity was 94.5% (95% CI, 94.4–94.6) [79].

On the other hand, 13 studies were included to evaluate the efficiency of the 99^th^ percentile cutoff in CHD detection, which included 180619 fetuses [17, 19-21, 69, 86, 88-90, 94, 97-99]. Of these fetuses, only 441 presented a major CHD [17, 19-21, 69, 86, 88-90, 94, 97-99]. The associated sensitivity was 19,5% (95% CI, 15.9–23.5), and the specificity was 99.1% (95% CI, 99.1–99.2) [79].

The results of these studies indicated that 61% of the fetuses diagnosed with CHDs presented an NT within the reference values, 16% of the fetuses had an NT value above the 95^th^ percentile, but below the 99^th^ percentile, and in the last 23% of fetuses, the NT value surpassed the 99^th^ percentile [79].

In short, the most recent data about fetuses with a NK have shown that the 95^th^ percentile cutoff allows for the diagnosis of 1 fetus with CHD out of 48 suspected fetuses, while the cutoff of the 99^th^ percentile allows for the diagnosis of 1 in 19 suspected fetuses [80]. On the other hand, the CHD risk is 20 times higher in fetuses with an NT value above the 99^th^ percentile [79].

### 5.3 Studies in which NT was Measured only by FMF-certified Operators (1999 Until 2013)

In an attempt to overcome the heterogeneity underlying the existing studies, studies in which NT was measured only by FMF-certified operators were evaluated [79]. The truth is, even so, a large heterogeneity was seen [79].

Through the collection of data pertaining to 11 studies, with a grand total of 105730 fetuses, it was seen that the proportion of fetuses with major CHDs was 2.6:1000 [20, 69, 86, 89-96]. All these studies used only the 95^th^ percentile as a cutoff, with the exclusion of all studies with cutoffs expressed in absolute values [20, 69, 86, 89-96]. The sensitivity obtained was 45.6% (95% CI, 39.6–51.7), and the specificity was 96.6% (95% CI, 95.1%-97.6%) [80].

8 additional studies were analyzed to study these parameters when the cutoff was the 99^th^ percentile, in which one CHD in 295 fetuses was identified, for a total of 118463 fetuses [20, 69, 86, 89-91, 95]. The sensitivity obtained was 21.0% (95% CI 16,5–26,1), and the specificity was 99.2% (95% CI, 99,2–99,3) (Table **3**) [79].

## 6 NUCHAL TRANSLUCENCY AND MAJOR CONGENITAL HEART DEFECTS IN FETUSES WITH NORMAL KARYOTYPE

Most studies that focused on this topic showed that increased NT can be related to the presence of several CHDs, with distinct hemodynamic consequences [32, 100]. On the other hand, there are also reports of some CHDs in which the prevalence did not show any significant differences between fetuses with or without increased NT [32, 100]. As examples, we have ventricular septal defects (VSD) and hypoplastic left heart syndrome [32, 100]. In addition, and independent of the NT value, fetuses with these CHDs presented a similar cardiothoracic ratio [32, 101]. Similarly, in cases of fetuses with isolated VSD, there were also no significant differences in ejection fraction [32, 101].

The way that different studies report the results relative to each specific type of CHD varies greatly, with some opting to address them individually and others defining groups according to structural or functional similarities. Because we think it is easier to understand grouped evaluations, we considered 7 groups, adopting a strategy based on the division made by *Makrydimas et al.* [78]: Laterality defects (LDs), right heart defects (RHDs), septal defects (SDs), left heart defects (LHDs), outflow tract abnormalities (OTAs), complex abnormalities and other abnormalities [16, 78, 102]. LDs include left and right atrial isomerism [78, 103]. RHDs include Ebstein’s anomaly, tricuspid atresia, tricuspid valve dysplasia, pulmonary atresia with intact ventricular septum, and pulmonary valve stenosis [78, 103-105]. Examples of SDs include atrioventricular septal defects with normal situs [78, 102, 105], persistent foramen ovale [101] and VSD [78, 102-104]. For LHDs, examples include aortic valve stenosis, aortic atresia with or without mitral atresia, coarctation of the aorta with or without a ventricular septal defect and hypoplastic left heart [78, 102-105]. OTAs are represented by absent pulmonary valve syndrome, common arterial trunk, transposition of the great arteries with or without a VSD and tetralogy of Fallot with or without atresia [78, 102-105]. Defects such as double inlet ventricles, atrioventricular to ventriculoatrial discordance and double outlet right ventricle belong to the complex abnormalities group [78, 102-104]. In addition, we can also detect cardiomyopathy [78, 98, 103], Wolff-Parkinson-White syndrome [98], and anomalous pulmonary venous drainage [78, 103], among other defects [103].

A study found that NT values of fetuses with CHDs have a bimodal distribution, with a value of 3.5 mm being the one that is associated, overall, with fewer cases [78]. As exceptions, we can highlight LHDs, in which the most inferior point of the respective graphical curves is closer to 3 mm [104]. As discussed before, the best NT cutoff for the early detection of CHDs is not yet agreed on [78]. However, it is widely known that fetuses with NT values above the 99^th^ percentile require a more detailed evaluation of the cardiovascular system by a fetal cardiologist using an echocardiogram [78]. In turn, these data are not as robust in the case of fetuses with NT values between the 95^th^ and 99^th^ percentiles [78]. With this in mind, we expect that there is a larger rate of CHD detection in the fetuses that were first mentioned in comparison to the other fetuses [78]. Therefore, we conclude that this potential bias is not in agreement with the lowest recorded value of CHDs for an NT value of 3.5 mm [78].

On the other hand, older studies support the idea that the association between CHDs and increased NT shown on the first-trimester ultrasound is better established for defects at the AA level [32]. Some studies have concluded that NT values under 3.5 mm SD are the most prevalent [103, 106, 107] and can correspond to approximately 20% of all detected CHDs [101]. For NT values above this value, no specific type stood out, presenting similar distributions [103, 107].

It has also been described that some CHDs present variability in their prevalence, depending on the fetal karyotype [78]. Fetuses with NK are more affected by LDs and RHDs [78]. On the other hand, they seem to be less susceptible to the presence of SDs [78]. Beyond this, and for the same CHD, NT values are generally lower in these fetuses than in fetuses with abnormal karyotypes [78].

In general, the studies showing that NT values measured in karyotypically normal fetuses with the most diverse CHDs are relatively similar [78]. Any differences seen, although not statistically significant, can be completely random, taking into consideration the reduced number of fetuses included for each CHD [80]. In this way, we can say that it is a unanimous conclusion in the current scientific evidence that the detection of different major CHDs does not seem to correlate with specific NT values [9, 16, 32, 78, 99, 108, 109]. This makes us question the physiopathological mechanisms that result in increased NT even more, given that fetal hemodynamic abnormalities do not explain, in an isolated way, why fetuses with the same CHD can present different NT values [33]. Simpson *et al.* also evaluated the possibility that fetuses with increased NT could have a higher rate of specific types of CHDs that would make them more susceptible to insufficient uteroplacental circulation [32]. This hypothesis was not, however, supported by any study [32]. Therefore, CHD severity is the main determinant of fetal prognosis, not the magnitude of the NT increase, in an isolated way [78].

## 7 DISCUSSION

Little is known about the physiological mechanisms that serve as the basis for NT. On the other hand, in the last few years, several hypotheses to explain the physiopathological mechanism of increased NT have been reported. Despite this, the physiopathology is not yet completely understood. It is likely that increased NT has a multifactorial etiology, likely resulting from cardiovascular abnormalities, lymphatic abnormalities, a deficient EM, head and neck venous congestion, infections, anemia, hypoproteinemia and fetal hypokinesia/akinesia. The first three are the ones with the most evidence.

The detection of increased NT may not have any impact on the fetus but should indicate the possibility of an APO. There are two pathologies that are strongly associated with increased NT: T21 and the presence of CHD. However, another CA may be present, and the risk is directly proportional to the magnitude of the NT increase. On the other hand, fetuses with increased NT present a relative risk of having a CHD that is 6.6 times higher and a risk that is at least 10 times higher for severe coronary disease. Other associated APOs are IUD, SAb, and a broad spectrum of SA, with increasing GSs being associated with increased NT. Regarding the gestation of MT, when one of the fetuses has an increased NT, the risk of TTTS is 3 times greater.

Currently, the best NT cutoff value for CHD detection is not clear. Despite the increase in resolution in ultrasounds and changes in the methods used to measure NT, studies still show heterogeneous results. According to scientific evidence up to 2013 relating to fetuses with NK, the cutoff for the 95^th^ percentile allows for the diagnosis of 1 fetus with a CHD in a total of 48, whereas the cutoff of the 99^th^ percentile allows the diagnosis of 1 in 19. Additionally, fetuses with an NT value above the 99^th^ percentile seem to have a 20 times higher risk of developing a CHD. Studies in which NT was measured exclusively by FMF-certified operators did not seem to be effective in reducing the heterogeneity of the results.

The CHDs that are found more frequently in fetuses with increased NT can be divided into 7 groups: LDs, RHDs, SDs, LHDs, OTAs, complex abnormalities and other abnormalities. NT in fetuses with CHDs seems to have a bimodal distribution, with the nadir generally being 3.5 mm. When energy is under that value, the SD group has the preponderance, with a prevalence of approximately 20%. For larger values, no other group is highlighted.

Notably, some CHDs present a prevalence that is independent of the NT value, such as interventricular communication and hypoplastic left heart syndrome.

The prevalence of some CHDs can also vary according to the fetal karyotype. Fetuses with NK are more affected by LDs and RHDs and less affected by SDs. In addition, we can see that, usually, the same CHD is associated with lower NT values in fetuses with NK.

Additionally, according to current scientific evidence, all the different CHDs, in general, do not display statistically significant differences for measured NT values, which means that it is impossible to say that one CHD is more likely than another simply based on the NT value.

With this review, we intended to clarify all the physiopathological mechanisms that lead to increased NT, identify the possible APO, explore, in greater detail, the strong association between increased CHD risk and NT, collect the results seen in the literature about the advantages and disadvantages of different NT cutoffs values in CHD detection and show that different NT values are not associated with specific types of CHDs. Further investigation is required to better understand the physiology that is the basis for NT, identify other APOs associated with increased NT, which may not have been identified yet due to the low number of cases, and determine a specific NT cutoff value that is unanimous and universal for CHD detection.

## CONCLUSION

In conclusion, the association between CHDs and increased NT is well established, but there is no consensus on the best NT cutoff point for CHD detection. There appear to be no significant differences among CHDs regarding measured NT values. Thus, the prognosis is mainly determined by CHD severity and not by NT thickness.

## Figures and Tables

**Table 1:** Comparison of the results obtained for the variables: Pooled true positive, pooled false-negative, pooled sensitivity and pooled specificity with different NT cutoffs, namely, the 95^th^ percentile (or 2.2-2.8 mm, or ≤ 3 mm) and 99^th^ percentile (or ≥ 3.5 mm, or between 3-5 mm).

Variable	95^th^ Percentile (or 2.2-2.8 mm, or ≤ 3 mm)	99^th^ Percentile (or 3-5 mm, or ≥ 3.5 mm)
Number of studies	8 [20, 22, 69, 81-85]	6 [20, 22, 69, 81, 83, 85]
Total number of fetuses	58492	49804
Fetuses with CHDs	162	139
Pooled true-positive	66	46
Pooled false-negative	96	93
Pooled sensitivity	37% (95% CI, 25%-51%)	31% (95% CI, 18%-49%)
Pooled specificity	96.6% (95% CI, 95.1%-97.6%)	98.7% (95% CI, 98.1%-99.2%)

**Table 2:** Comparison of the results obtained for the variables: Pooled sensitivity and pooled specificity for different NT cutoffs, namely, the 95^th^ percentile and the 99^th^ percentile.

Variable	NT value > 95^th^ Percentile	NT value > 99^th^ Percentile
Number of studies	17 [17, 19, 20, 62, 17, 86-98]	13 [17, 19-21, 69, 86, 88-90, 94, 97-99]
Total number of fetuses	151369	180619
Fetuses with CHDs	427	441
Pooled sensitivity	44.4% (95% CI, 39.5–49.5)	19.5 (95% CI, 15.9–23.5)
Pooled specificity	94.5% (95% CI, 94.4–94.6)	99.1% (95% CI, 99.1–99.2)

**Table 3:** Comparison of the results obtained for the variables in studies in which NT was measured only by FMF-certified operators: Pooled sensitivity and pooled specificity for different NT cutoffs, namely, the 95^th^ percentile and the 99^th^ percentile.

Variable	NT value > 95^th^ Percentile	NT value > 99^th^ Percentile
Number of studies	11 [20,69,86,89-96]	8 [20,69,86,88-90,94,99]
Total number of fetuses	105730	118463
Fetuses with CHDs	274	295
Pooled sensitivity	45.6% (95% CI, 39.6–51.7	21.0% (95% CI 16.5–26.1)
Pooled specificity	96.6% (95% CI, 95.1%-97.6%)	99.2% (95% CI, 99.2–99.3)

## LIST OF ABBREVIATIONS

VSD: Ventricular Septal Defects
LDs: Laterality Defects
RHDs: Right Heart Defects
SDs: Septal Defects
LHDs: Left Heart Defects
OTAs: Outflow Tract Abnormalities
PPV: Positive Predictive Value
